# Prediction of portal dosimetry quality assurance results using log files-derived errors and machine learning techniques

**DOI:** 10.3389/fonc.2022.1096838

**Published:** 2023-01-13

**Authors:** Kah Seng Lew, Clifford Ghee Ann Chua, Calvin Wei Yang Koh, James Cheow Lei Lee, Sung Yong Park, Hong Qi Tan

**Affiliations:** ^1^ Division of Radiation Oncology, National Cancer Centre Singapore, Singapore, Singapore; ^2^ Oncology Academic Clinical Programme, Duke-NUS Medical School, Singapore, Singapore

**Keywords:** machine learning, patient specific quality assurance, portal dosimetry, log file, radiotherapy

## Abstract

**Objective:**

This work aims to use machine learning models to predict gamma passing rate of portal dosimetry quality assurance with log file derived features. This allows daily treatment monitoring for patients and reduce wear and tear on EPID detectors to save cost and prevent downtime.

**Methods:**

578 VMAT trajectory log files selected from prostate, lung and spine SBRT were used in this work. Four machine learning models were explored to identify the best performing regression model for predicting gamma passing rate within each sub-site and the entire unstratified data. Predictors used in these models comprised of hand-crafted log file-derived features as well as modulation complexity score. Cross validation was used to evaluate the model performance in terms of R^2^ and RMSE.

**Result:**

Using gamma passing rate of 1%/1mm criteria and entire dataset, LASSO regression has a R^2^ of 0.121 ± 0.005 and RMSE of 4.794 ± 0.013%, SVM regression has a R^2^ of 0.605 ± 0.036 and RMSE of 3.210 ± 0.145%, Random Forest regression has a R^2^ of 0.940 ± 0.019 and RMSE of 1.233 ± 0.197%. XGBoost regression has the best performance with a R^2^ and RMSE value of 0.981 ± 0.015 and 0.652 ± 0.276%, respectively.

**Conclusion:**

Log file-derived features can predict gamma passing rate of portal dosimetry with an average error of less than 2% using the 1%/1mm criteria. This model can potentially be applied to predict the patient specific QA results for every treatment fraction.

## 1 Introduction

Patient-specific quality assurance (PSQA) is an important part of radiotherapy treatment for cancer patients. With advances in treatment modalities and linear accelerators technology, a more rigorous PSQA programme or method is required to ensure that the increasingly complex treatment plans are correctly delivered as planned (1). In recent years, the numbers of works involving the application of machine learning (ML) to PSQA has been increasing in order to better model the complex relationship between mechanical and dosimetric parameters (2). PSQA is usually performed by comparing actual dose (either absolute dosimetry or relative dosimetry) using dose measurement device with the planned dose. EPID with portal dosimetry has been widely used for PSQA as it is able to reduce the workload of quality assurance while maintaining the quality of measurement (3). EPID detector also has an additional advantage of being a high-resolution detector with resolution being determined by the pixel spacing, which is usually less than 500 microns. Without the need for using film dosimetry protocol or additional equipment set-up, portal dosimetry can be done efficiently and accurately once it has been commissioned (4).

The analysis of log file has been suggested by various studies as a complement to measurement based PSQA (5–7). Sun et al. (6) concluded that machine log file has the ability to identify errors during beam delivery and that errors in individual MLC leaf can also be detected instead of being masked up by a measurement-based approach. In the paper by Szeverinski et al. (7) LINACWatch^®^ (Qualiformed, La Roche-sur-Yon, FRA) has the ability to detect small delivery errors and that MLC shift errors is more sensitive to log files analysis compared to ArcCHECK^®^ detector (Sun Nuclear, Melbourne, FL) measurement. In this work, we will attempt to predicting the gamma passing rate of portal dosimetry using log file information with four different ML algorithms. The modelling involving log file and portal dosimetry has not been reported in the literature before at the point of writing the manuscript. Features input into the ML algorithm will be extracted from the log file as we will be focusing on actual deviations from planned machine parameters as well as complexity metrics. This model will eventually serve as a virtual QA to be implemented by collecting log file from each completed fraction. Even though the log file data contain information of the mechanical and monitor unit (MU) errors which could by itself, be used for PSQA, there are several rationales behind our study for connecting the log file errors to PSQA results. First, the gamma passing rate is a familiar scalar quantity and most centres have developed years of clinical experience with this metric and understand what kind of threshold value is indicative of a poor plan (with disagreement between planned and delivered dose). Second, there is currently no consensus on how to best process the multi-parameters log file data to indicate a failing plan especially with the many confounding variables such as treatment modality (VMAT vs IMRT), number of treatment fields, beam energies and dose prescription. Thirdly, our study aims to understand the correlation between machine errors which are found in the log file to familiar gamma passing rate in PSQA to help identify the root cause for failure in PSQA. Lastly, replacing the actual portal dosimetry measurement with our proposed “virtual QA” can reduce wear and tear on electronic portal imaging device (EPID) detectors which in our experience, can be expensive to replace and may incur significant downtime.

## 2 Materials and methods

### 2.1 Data and measurement

In this study, trajectory log files were collected from a single Varian TrueBeam (Varian Medical Systems, Palo Alto, CA) equipped with Millennium 120 MLC retrospectively over a period of six months from January 2021 to June 2021. The inclusion criteria are SBRT and VMAT treatment as routine pre-treatment PSQA are compulsory in this hypofractionated treatment in our centre. The data are stratified into three different sites - prostate, spine, and thorax. Patient specific QA is performed using the aS1200 EPID and quantified using gamma analysis with Portal Dosimetry module (Eclipse ver. 13.6). The EPID has an area of 43 x 43 cm^2^ and is set at a distance of 140.0 cm away from the beam source (also known as Source to Imager Distance). This distance is chosen as it is the distance calibrated for clinical use. In the Eclipse’s portal dosimetry module, a low-dose threshold of 10% was included when calculating the global gamma passing rate (GPR) for PSQA. This will ignore the large volume of dose points that lies in the low-dose regions that might inflate the global GPR (1). For the purpose of this study, gamma analysis was performed using the 2%/2mm, 2%/1mm, 1%/2mm and 1%/1mm criteria in absolute dose and results were collect using field by field approach (8). The composite result can be obtained by summing the portal doses per field; this can be easily performed within the module.

Log files from TrueBeam are output during every treatment or QA with both planned and actual machine parameters recorded. The log files consist of a time series of machine parameters recorded every 20 ms.

### 2.2 Handcrafted features calculated from log file data

**Table 1** shows the 13 features derived from the log file data were used for ML modelling to predict for the global gamma passing rate. They consist of average root mean square errors (RMSE) of various VMAT delivery parameters with an additional plan complexity metric, Modulation Complexity Score (MCS) (9) included in the features to identify how MLC complexity of the plan affects the GPR of a treatment plan.

**Table 1:** Table of handcrafted features from log file.

Derived Features	
Field Opening RMSE	MLC Acceleration RMSE
Gantry Position RMSE	Weighted MLC Position RMSE
Gantry Velocity RMSE	Weighted MLC Velocity RMSE
Gantry Acceleration RMSE	Weighted MLC Acceleration RMSE
MLC Position RMSE	MU/Arc
MLC Velocity RMSE	MCS
Dose Rate	

To calculate the RMSE of machine parameters, a general formula is used:


(1) $$\bar{RMSE}\;=\;\sqrt{\frac{1}{N}\sum_{i=0}^{N}{\left(x_{i}-{\hat{x}}_{i}\right)}^{2}},$$

*x*_*i*_ is the planned value for the parameter, ${\hat{x}}_{i}$ is the actual value for the parameter (gantry angle and MLC positions) and N is the number of time sampled point. In weighted MLC RMSE parameters, they are weighted using the ratio of the MU at each time point to the total MU, followed by multiplying the MLC RMSE parameters between each time sample point. The formula is as follow:


(2) $$\bar{RMSE_{mu}}\;=\;\sqrt{\frac{1}{N}\sum_{i=0}^{N}{\left(\frac{mu_{j}}{\sum_{j=0}^{N}mu}x_{j}-\frac{{\hat{mu}}_{k}}{\sum_{k=0}^{N}\hat{mu}}{\hat{x}}_{k}\right)}^{2}},$$

where *x*_*i*_ is the planned value for the parameter, ${\hat{x}}_{i}$ is the actual value for the parameter, *mu*_*j*_ and ${\hat{mu}}_{k}$ are the actual MU and planned MU respectively and N is the total sampling points. A single RMSE is then calculated.

Correlation between different features and gamma passing rates are analysed using Spearman’s correlation. The correlations are calculated for different treatment sites individually as well as all treatment site together. A two-sided *P-value* of less than 0.05 is regarded as statistically significant in this study.

### 2.3 Machine learning model

**Figure 1:**
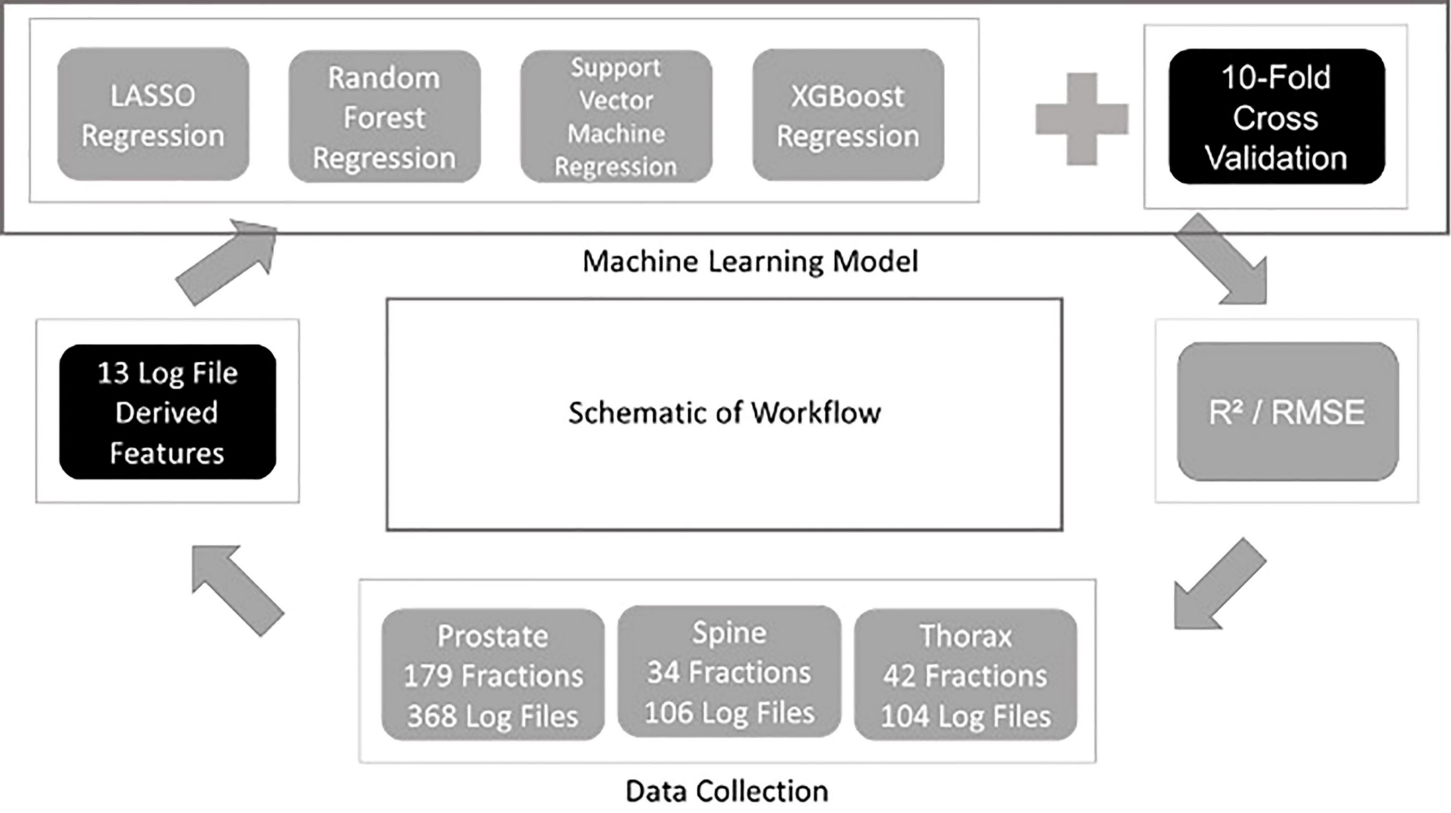
Schematic of workflow for this study.

Four different regression ML algorithms are used in this study: - LASSO Regression, Support Vector Machine Regression, Random Forest Regression and XGBoost Regression. Regression is a technique used for relating the relationship between features and a continuous outcome. In this study, the features extracted from the log files were used to train each ML model followed by the prediction of the outcome which is the GPR for each plan. The use of ML model in regression problem enables the modelling of the complex relationship between the features and the GPR. The model building and evaluation pipelines are shown in **Figure 1**. The same pipeline was utilised for all algorithms and the model is evaluated using a ten-fold cross validation method. In every fold, 10% of the data from all treatment sites was assigned as a hold-out set (testing set) while 90% of them were used as the train set. For each fold, the hyperparameters are optimised using another inner nested cross validation within the training set. The optimal model in this fold is used to predict the GPR in the hold-out set. R^2^ and RMSE evaluated on all the hold-out sets from the 10 folds are used to quantify the generalizability of the model which completes the model validation phase. A ten-folds cross validation approach has an advantage of lesser bias compared to a single train-test split for model validation. R^2^ and RMSE metrics are also used to determine the best performing ML algorithm for predicting GPR. The results obtained from the 10 different folds are then shown together with their standard deviation according to their training models and data stratification. In XGBoost Regression, a F score is calculated to obtain the importance of the derived features (10). In this work, all models are developed using Python programming language. Pylinac (11) is used to extract data from log files for calculations and scikit-learn (12) library for model building and evaluation.

After the model training and evaluation phase, we will need a final model that can be tested clinically. In data science, one of the approaches of arriving at the final deployed model is by training the ML pipeline on the entire datasets (include all train-validation-test data). The performance of each model on the current dataset is quantified again for completeness. It is important to note the performance of this model in the overall dataset is not part of the model evaluation process (since model evaluation must always be quantified on a hold-out set which is unseen to the model) and serve just as a soundness check for the final model.

## 3 Results

### 3.1 Data and measurement

**Figure 2:**
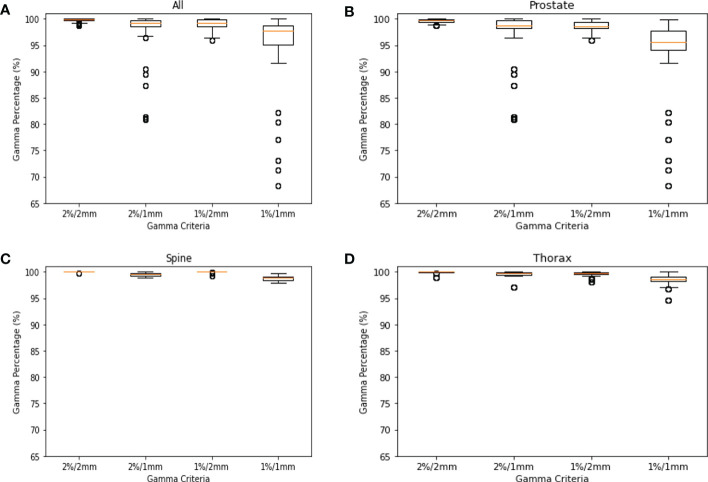
GPR Plots for Log File. **(A)** GPR of all log files for 2%/2mm, 2%/1mm, 1%/2mm & 1%/1mm. **(B)** GPR of prostate log files for 2%/2mm, 2%/1mm, 1%/2mm & 1%/1mm. **(C)** GPR of spine log files for 2%/2mm, 2%/1mm, 1%/2mm & 1%/1mm. **(D)** GPR of thorax log files for 2%/2mm, 2%/1mm, 1%/2mm & 1%/1mm.

This study was approved by the SingHealth institutional review board. A total of 578 log files collected was used for modelling in the study. They consist of 368 log files from prostate, 106 log file from spine and 104 log files from thorax. In **Figure 2**, the GPR for each site are plotted to show the distributions. Across all plots, 1%/1mm has the biggest interquartile range (IQR) while 2%/2mm has the smallest IQR. In **Figure 2C**, we can see that 2%/2mm and 1%/2mm has a spread so small that the IQR cannot be determined. A small spread is not ideal as it is challenging to derive a robust cut-off value for “passing” plan and could make it harder to differentiate the quality of the plan (13).

### 3.2 Correlation between features

**Figure 3:**
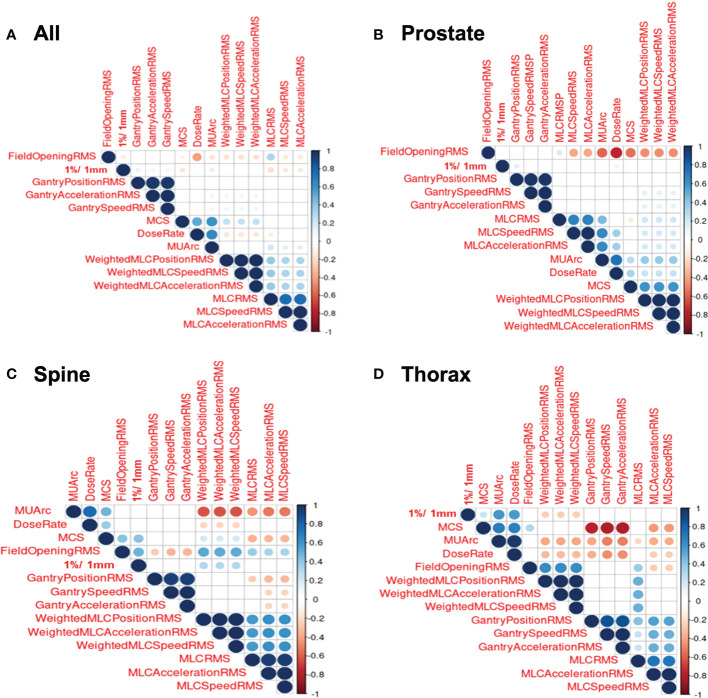
Correlation Plots for Log File. **(A)** Correlation plot for all log files. **(B)** Correlation plot for prostate log files. **(C)** Correlation plot for spine log files. **(D)** Correlation plot for thorax log files.

**Figure 3** shows the correlation plots for features calculated for all treatment sites, prostate, thorax and spine. Only the GPR calculated at 1%/1mm criteria was included (here and in further modelling) as the GPR for other criteria are not significantly correlated with the features. Looking at the correlation plot for all treatment sites in **Figure 3A**, a weak positive correlation is observed between gantry related RMSE and the 1%/1mm GPR while MCS, MU/arc and MLC related RMSE are also weakly negatively correlated. In **Figure 3B**, for prostate treatment, a weak positive correlation was observed between gantry position RMSE and dose rate with 1%/1mm GPR. In **Figure 3C**, a positive correlation between 1%/1mm GPR and weighted MLC related RMSE, field opening and MCS can be observed for spine treatment site. In **Figure 3D** for thorax treatment site, there is a negative correlation between 1%/1mm GPR and weighted MLC related RMSE while having a positive correlation with MCS, dose rate and MU/arc.

### 3.3 Machine learning model

**Table 2** shows the overall results of R^2^ across all the 10-fold cross validation sets. LASSO and XGBoost regression represent the worst-performing and best-performing ML algorithm respectively in terms of goodness of fit. For models stratified according to treatment site, the modelling of spine treatment site has the worst fit. Similarly, **Table 3** shows the overall result of RMSE across all the 10-fold cross validation sets with both mean and standard deviation. Again, XGBoost regression has the smallest RMSE amongst all the ML algorithms used.

**Table 2:** R^2^ values across all 10-fold for each model.

R^2^	All	Prostate	Spine	Thorax
LASSO Regression	0.121 + 0.005	0.024 + 0.009	-19.904 + 1.971	-0.853 + 0.101
SVM Regression	0.605 + 0.036	0.537 + 0.046	-0.681 + 0.971	0.700 + 0.149
Random Forest Regression	0.940 + 0.019	0.933 + 0.022	0.230 + 0.370	0.831 + 0.118
XGBoost Regression	0.981 + 0.015	0.979 + 0.018	0.602 + 0.577	0.965 + 0.017

**Table 3:** RMSE values across all 10-fold for each model.

RMSE	All	Prostate	Spine	Thorax
LASSO Regression	4.794 ± 0.013	5.801 ± 0.027	2.417 ± 0.116	1.674 ± 0.045
SVM Regression	3.210 ± 0.145	3.990 ± 0.193	0.651 ± 0.217	0.641 ± 0.170
Random Forest Regression	1.233 ± 0.197	1.502 ± 0.249	0.452 ± 0.108	0.474 ± 0.148
XGBosst Regression	0.652 ± 0.276	0.777 ± 0.372	0.267 ± 0.200	0.217 ± 0.062

**Figure 4:**
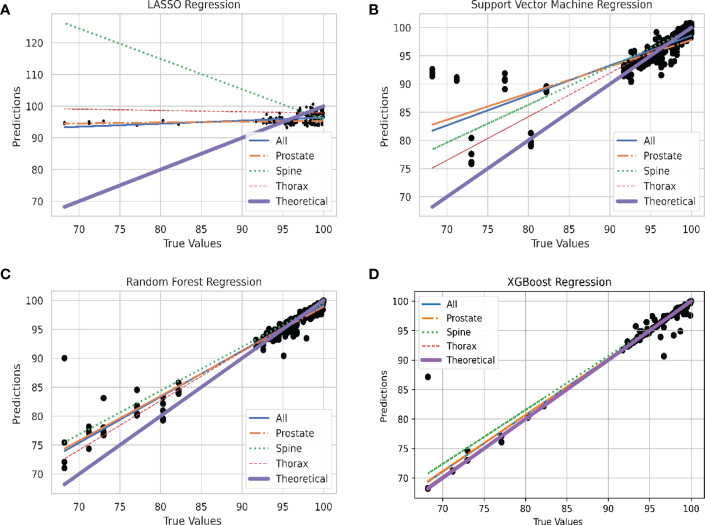
Linear Plot of Predicted and Theoretical GPR. **(A)** Linear plot of each site stratification vs. actual GPR for LASSO Regression model. **(B)** Linear plot of each site stratification vs. actual GPR for Support Vector Machine Regression model. **(C)** Linear plot of each site stratification vs. actual GPR for Random Forest Regression model. **(D)** Linear plot of each site stratification vs. actual GPR for XGBoost Regression model.

In **Figure 4**, the predicted values of each model are plotted against the true values using the final model. They are divided according to unstratified treatment site, prostate, spine and thorax treatment site. The ideal model is also plotted and is shown as the theoretical 45-degree line where the predicted values should be the same as the true value. Generally, all the models are able to produce the correct trend (positive gradient) except for the spine plans in LASSO regression model which yields a negatively sloped best fit line. XGBoost algorithm has the closest plot to the theoretical best fit line when compared across all the four different plots.

**Figure 5:**
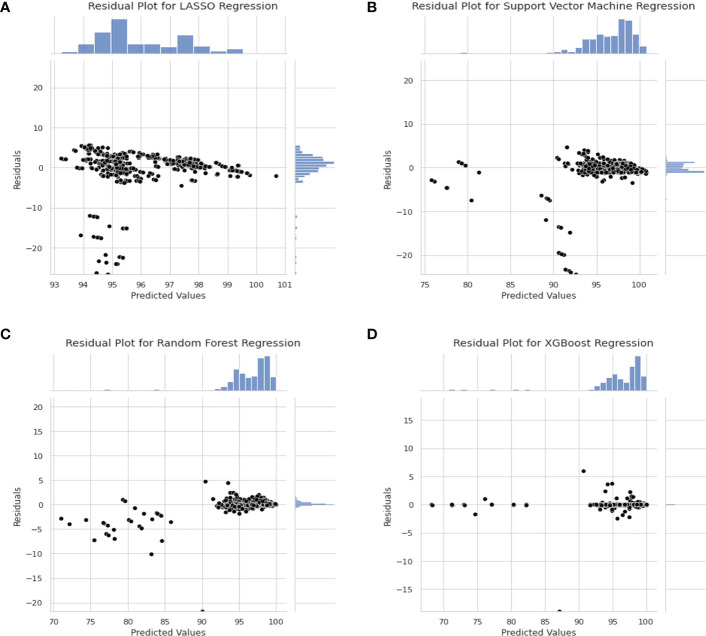
Residual Plot for Different Model. **(A)** Residual plot for LASSO Regression Model. **(B)** Residual Plot for Support Vector Machine Regression. **(C)** Residual Plot for Random Forest Regression. **(D)** Residual plot for XGBoost regression model.

Residual plots are shown in **Figure 5** to show the distribution of the difference between true and predicted values. In this case, residuals from all models shows a Gaussian-like distribution for the residuals other than Support Vector Machine regression. The distribution of the residuals in **Figure 5D** are very narrow which results in a “Dirac Delta-like” distribution in the figure. In **Figure 5B**, the predicted values above 100% are outliers in the residual plots. Also, in **Figures 5A–C** the distribution of residual is biased towards the positive values.

**Figure 6:**
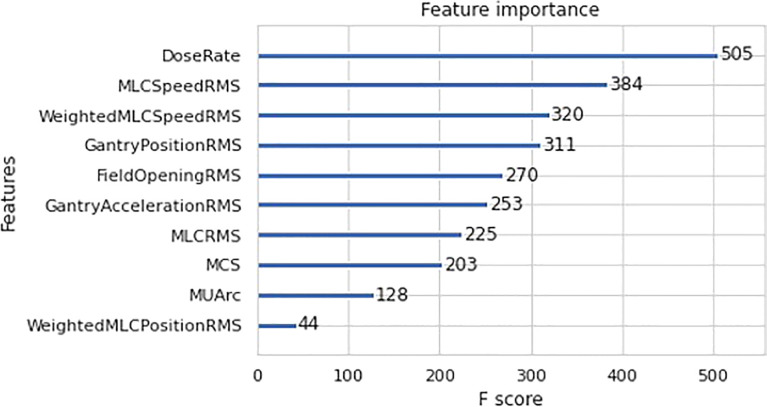
Importance F-Score for XGBoost Regression.

Further analysis was done to determine the individual feature contribution towards GPR prediction for the best model - XGBoost regression. The feature importance plot for XGBoost is shown in **Figure 6** to determine the most important predictor in the model. In this case, dose rate was found to be the most significant predictor in the final XGBoost model.

## 4 Discussion

### 4.1 Correlation

A general observation seen across all the correlation heat maps in **Figure 3** was that gantry related RMSE has a weak or zero correlation with 1%/1mm GPR across all the four different stratifications. This is essentially due to the perpendicular composite dose measurement method where the detector is always moving together with the beam (1).

Looking at MLC related RMSE, most correlations across all four stratifications are found to be negatively correlated with GPR which are to be expected as a larger RMSE value would means that a larger error is present during beam delivery and thus, a lower GPR will be obtained. However, there is an anomaly for spine correlation. This could possibly be attributed to the large variability in the selected spine treatment plan as the treatment areas span C1 in the cervical vertebrae all the way to S5 in the sacrum. This causes a large variation in the treatment plan as different organ-at-risk will needs to be avoided for different location along the spine. We reckon a better approach to achieving higher performance in spine plan will be to further stratify the plans based on the spine positions. This, however, cannot be done currently in our datasets as there are insufficient log files within the spine site to accommodate further stratification, which could lead to an unreliable model with large prediction interval.

Focusing on the correlation involving field opening RMSE, all treatment sites show a negative correlation with GPR, spine treatment site shows a positive relationship while the last two stratifications show no significant correlation. MCS’s correlation with GPR also shows similar trend as field opening RMSE. This can be attributed to the field opening being one of the factors in the calculation of MCS. Overall, no conclusive correlation is observed between MCS and GPR. This agrees with the paper by M. C. Glenn et al. which mention complexity metrics have limited predictive utility in assessing plan performance (14).

### 4.2 Machine learning model

XGboost regression was found to be the best performing ML model for predicting GPR. It has a R^2^ value of 0.981 ± 0.015 which indicates a good fit when compared to a theoretical perfect value of 1. The RMSE also shows a value of (0.652 ± 0.276)%. XGBoost regression shows the lowest average RMSE value across the 10-folds which agrees with the R^2^ results in **Table 2**.

From **Tables 2**, **3**, we can see that LASSO regression and support vector regression do not perform as well in terms of R^2^; spine plan even shows a negative R^2^ value. In LASSO regression, the relationship between the variables and response are assumed to be linear which might not always be the case. On the other hand, support vector machine does not filter the features that are input into the model. This might result in introducing noise into the model which affects the decision model.

In the plot of predicted against true values of GPR in **Figure 4**, all the models are able to produce the expected relation between the predicted and true values of GPR except for spine treatment plans in LASSO model. For LASSO regression, a single point of outlier which predicted a value of above 100% for GPR can be seen. This might have resulted in an anomalous negative trend in the spine treatment site. The best performing model, XGBoost regression, has the greatest number of points lying close to the line which accounts for the high R^2^ value and also low RMSE value where data points are on average closer.

In the residual plot in **Figure 5**, the residuals of LASSO regression, Support Vector Machine regression and Random Forest regression is skewed towards the positive value which indicates that the model predicts a higher value than actual. This is not ideal as the model favours false negative. In clinical setting, false positive is preferred over false negative as it is better to predict a lower GPR for “passing plan” than a higher GPR for “failing plan”.

In the case of feature importance for XGBoost regression, dose rate is found to be the strongest predictor of GPR in the model. This can be explained by examining the correlation plot in **Figure 3A** which shows a highly statistically significant positive correlation between dose rate and MU per arc. The high MU is indicative of higher leakage and could lead to a lower GPR. This is substantiated by the negative correlation between MUs per arc and the 1%/1mm GPR in **Figure 3A**. This result agrees with the work by Wu et al. which mention that the higher the number of fields and MUs in each field will affect the treatment efficiency and quality assurance passing rate (15). Higher MU also implies relatively smaller segments and higher modulation in treatment plans.

There are several limitations which can be found in this study. In ML modelling, the final model is commonly tested for generalisability across data. In this case, external testing data, which is absent, should be used for evaluation of the model. Also, although there is a large number of log files used in this study (when comparing across the sample size used in PSQA-related ML studies), the number of unique patients is limited. Future study involving a larger patient sample size will need to be conducted. Also in this study, GPR is obtained using only portal dosimetry method. Despite the ability of portal dosimetry to identify various modes of delivery errors (16), there are other complementary PSQA tools in the commercial market. This work can be potentially extended to examine the prediction of GPR from other PSQA tools.

## 5 Conclusion

In this study, ML is able to predict the familiar GPR metric of treatment plans using log files. This can be helpful as PSQA could potentially be expanded to all treatment fractions and allows physicist to monitor LINAC consistency throughout treatment and intervene when needed. The reduction of wear and tear of the EPID can also saves cost and reduce downtime of machines.

## Data availability statement

The original contributions presented in the study are included in the article/supplementary material. Further inquiries can be directed to the corresponding author.

## Author contributions

Study conception and design: HT. Data acquisition and analysis: KL, CK, and CC. Data interpretation: All authors. Obtained funding: HT. Study supervision: HT, JL, and SP. Drafting of manuscript: KL and HT. Approval of final manuscript: All authors.
